# Global Prevalence of Antibiotic-Resistant *Burkholderia pseudomallei* in Melioidosis Patients: A Systematic Review and Meta-Analysis

**DOI:** 10.3390/antibiotics14070647

**Published:** 2025-06-25

**Authors:** Jongkonnee Thanasai, Sa-Ngob Laklaeng, Supphachoke Khemla, Khonesavanh Ratanavong, Moragot Chatatikun, Jitbanjong Tangpong, Wiyada Kwanhian Klangbud

**Affiliations:** 1 Faculty of Medicine, Mahasarakham University, Mahasarakham 44000, Thailand; jongkonnee@msu.ac.th; 2 School of Allied Health Sciences, Walailak University, Nakhon Si Thammarat 80160, Thailand; sumoun2528@gmail.com (S.-N.L.); moragot.ch@wu.ac.th (M.C.); rjitbanj@wu.ac.th (J.T.); 3 Division of Infectious Diseases, Department of Internal Medicine, Nakhon Phanom Hospital, Nakhon Phanom 48000, Thailand; sup.mednkp@gmail.com; 4 Savannakhet Health Science College, Savanakhet 13000, Laos; khonesavanh1212@gmail.com; 5 Medical Technology Program, Faculty of Science, Nakhon Phanom University, Nakhon Phanom 48000, Thailand

**Keywords:** *Burkholderia pseudomallei*, melioidosis, antimicrobial resistance, antibiotic resistance surveillance, antimicrobial susceptibility testing, public health

## Abstract

**Background**: *Burkholderia pseudomallei*, the causative agent of melioidosis, is intrinsically resistant to multiple antibiotics, posing substantial challenges for treatment. Reports of acquired resistance are increasing, underscoring the need for global surveillance. **Objective**: This systematic review and meta-analysis aimed to determine the global prevalence of antibiotic-resistant *B. pseudomallei* isolated from human clinical cases, with a focus on regional differences and variations in antimicrobial susceptibility testing methods. **Methods:** We systematically searched PubMed, Scopus, and Embase for studies reporting resistance in clinical *B. pseudomallei* isolates, following PRISMA guidelines. Pooled resistance rates to 11 antibiotics were calculated using a random-effect model. Subgroup analyses were performed based on geographical region and testing methodology (MIC vs. disk diffusion). **Results:** Twelve studies comprising 10,391 isolates were included. Resistance rates varied across antibiotics, with the highest pooled resistance observed for tigecycline (46.3%) and ciprofloxacin (38.3%). Ceftazidime (CAZ) and trimethoprim–sulfamethoxazole (SXT), commonly used first-line agents, showed resistance rates of 5.3% and 4.2%, respectively. Subgroup analyses of CAZ and SXT revealed significantly higher resistance in studies from Asia compared to Australia and America (*p* value < 0.0001). Disk diffusion methods tended to overestimate resistance compared to MIC-based approaches, which revealed non-significant differences for CAZ (*p* value = 0.5343) but significant differences for SXT (*p* value < 0.0001). **Conclusions:** Antibiotic resistance in *B. pseudomallei* exhibits regional variation and is influenced by the susceptibility testing method used. Surveillance programs and standardized antimicrobial susceptibility testing protocols are essential to guide effective treatment strategies and ensure accurate resistance reporting.

## 1. Introduction

*Burkholderia pseudomallei* is a Gram-negative, motile, facultative intracellular bacterium endemic to soil and surface water in tropical and subtropical climates, especially in Southeast Asia and Northern Australia [1]. It is the causative agent of melioidosis, a serious and often fatal infectious disease affecting both humans and animals. Human infection typically occurs via direct contact with contaminated soil or water, inhalation of dust particles, or ingestion of contaminated sources [2].

Melioidosis presents with diverse clinical manifestations, ranging from asymptomatic colonization to acute septicemia and chronic suppurative disease [2]. The acute form, typically emerging 1–2 weeks post-exposure, often presents with high fever, malaise, myalgia, respiratory symptoms, gastrointestinal distress, and, in severe cases, septic shock and multi-organ failure. Chronic melioidosis, more common in individuals with comorbidities such as diabetes mellitus or those with immunosuppressive conditions, may evolve over several weeks or months. It is characterized by prolonged fever, weight loss, fatigue, and pulmonary involvement. The disease often involves multi-organ systems, with abscess formation in the lungs, liver, spleen, and central nervous system being a hallmark feature. Latent infections may also occur, with potential reactivation following immunosuppression [3,4].

Therapeutic management of melioidosis remains challenging due to the organism’s intrinsic resistance to many commonly used antibiotics, including aminoglycosides and most penicillins [5,6]. Current treatment regimens are typically divided into two phases: an intensive phase, involving intravenous administration of ceftazidime or carbapenems (e.g., meropenem or imipenem), followed by a prolonged oral eradication phase using trimethoprim–sulfamethoxazole (SXT) or doxycycline [5,6]. While these regimens are generally effective, treatment failures and relapses remain common, often due to antimicrobial resistance or non-compliance.

In recent years, increasing reports of antibiotic-resistant *B. pseudomallei* strains have emerged, raising significant public health concerns [7,8,9,10,11,12,13,14,15,16,17,18]. Resistance to first-line agents such as ceftazidime and SXT has been documented, especially in endemic regions like Thailand and Malaysia. More alarmingly, elevated resistance to alternative agents like ciprofloxacin and tigecycline has also been observed, although these antibiotics are not typically part of standard treatment protocols. These findings underscore the urgent need for comprehensive and updated data on resistance patterns to inform clinical practice and public health policies.

A comprehensive overview of the global distribution of *B. pseudomallei* is essential to understanding the epidemiology of the disease. While melioidosis is primarily endemic in Southeast Asia and Northern Australia, cases have also been reported in other regions, including South America and parts of Africa [4]. Morbidity and mortality rates vary significantly by region, with higher incidence rates in tropical climates [1,3]. The case fatality rate for melioidosis is notably high, ranging from 20% to 50% in severe cases, particularly in patients with comorbidities [2]. The reproductive rate of *B. pseudomallei* in endemic areas is a critical factor in understanding disease transmission, and more research is needed to clarify these dynamics globally.

In addition to geographical variability, the methodology used for antimicrobial susceptibility testing (AST) significantly influences the detection and reporting of resistance. Common AST methods include broth microdilution (BMD), E-test, disk diffusion, and automated systems such as VITEK^®^ 2 (bioMérieux, Marcy-l'Étoile, France) and Sensititre^®^ (Thermo Fisher Scientific, MA, USA). Among these, MIC-based approaches like BMD are considered the gold standard due to their higher accuracy and reproducibility. However, resource limitations in many endemic regions have led to widespread reliance on disk diffusion, which may overestimate resistance due to methodological limitations, especially for antibiotics like SXT and tigecycline [12,15].

This systematic review and meta-analysis aim to provide a comprehensive and quantitative synthesis of the global prevalence of antibiotic-resistant *B. pseudomallei* in clinical settings. By examining data across various geographic regions and AST methods, this study seeks to clarify resistance trends, identify knowledge gaps, and guide the development of effective treatment strategies. The findings will support antimicrobial stewardship programs and promote the standardization of diagnostic practices for melioidosis, especially in high-burden countries.

## 2. Results

### 2.1. Search Results

**Figure 1:**
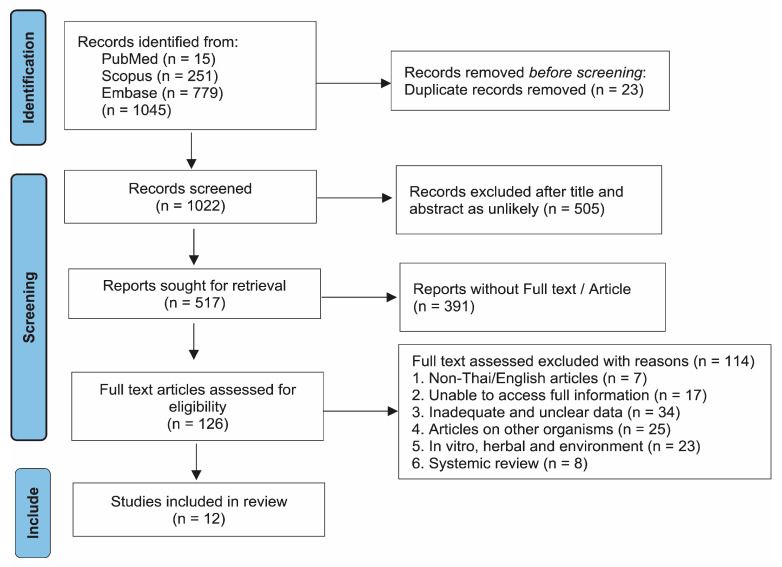
PRISMA flow diagram.

The PRISMA flow diagram illustrates the selection process for studies included in the meta-analysis on the prevalence of antibiotic drug-resistant *Burkholderia pseudomallei*, as shown in Figure 1. Initially, a total of 1045 records were identified from three databases: PubMed (15), Scopus (251), and Embase (779). After removing 23 duplicate records, 1022 studies remained for screening. At this stage, 505 records were excluded, as they were deemed unlikely to be relevant based on their titles and abstracts. Subsequently, 517 reports were sought for full-text retrieval, but 391 could not be accessed in full. Among the 126 full-text articles assessed for eligibility, 114 were excluded for various reasons, including non-English or non-Thai language (7),; lack of full information (17); inadequate or unclear data (34); focus on organisms other than *B. pseudomallei* (25); studies on in vitro, herbal, or environmental aspects (23); and systematic reviews (8). Ultimately, 12 studies [7,8,9,10,11,12,13,14,15,16,17,18] met all eligibility criteria and were included in the final meta-analysis.

### 2.2. Overview of the Included Studies

A total of 12 studies [7,8,9,10,11,12,13,14,15,16,17,18] were included in this meta-analysis, comprising 10,391 clinical isolates of *Burkholderia pseudomallei* from various countries, as shown in Table 1. The majority of the studies originated from Thailand (5 studies) [9,14,15,17,18], followed by Malaysia (3 studies) [7,12,16], the USA (1 study) [8], Australia (1 study) [11], Vietnam (1 study) [13], and China (1 study) [10]. Different antimicrobial susceptibility testing methods were used, including nine studies of minimum inhibitory concentration (MIC) via E-test [7,12], broth microdilution (BMD) [8,9,11,13,14,17], and automation-based such as VITEK 2^TM^ [9,10,17] and Sensititre^TM^ [13,17], as well as three studies of disk diffusion assays [15,16,18]. The characteristics of the included studies are shown in Table 1.

The antibiotic resistance patterns varied across studies and geographic locations. As illustrated in Table 1, ceftazidime (CAZ) resistance was observed in multiple studies, particularly in Hui et al. (2022) (3/45 resistant isolates; 6.7%) [10], Khosravi et al. (2014) (4/69 resistant isolates; 5.8%) [12], Jenney et al. (2001) (7/170 resistant isolates; 4.1%) [11], and Paveenkittiporn et al. (2009) (60/4019 resistant isolates; 1.5%) [15]. Trimethoprim–sulfamethoxazole (SXT) resistance was significantly high in studies from Thailand; for instance, Paveenkittiporn et al. (2009) [15] reported resistance in 1889 isolates (1889/4019 resistant isolates; 47%). Resistance to doxycycline (DOX) and chloramphenicol (CHL) was variable, while carbapenems, including imipenem (IPM) and meropenem (MEM), showed consistently low resistance rates, indicating their continued effectiveness. Amoxicillin/clavulanic acid (AMC) and amoxicillin (AMX) showed limited resistance, with a few cases reported by Ahmad et al. (2013) [7] and Khosravi et al. (2014) [12]. Additionally, piperacillin/tazobactam (TZP) resistance was observed in some studies but remained generally low.

Geographical variations in resistance patterns were evident. Studies from Thailand and Malaysia reported higher resistance rates to SXT and CAZ, while a study in the USA (Bugrysheva et al., 2021) [8] found notable resistance to aztreonam (ATM) and aztreonam/avibactam (AZA). In contrast, studies from China [10] and Vietnam [13] demonstrated low resistance rates across most of the tested antibiotics. These findings highlight the regional differences in antibiotic resistance and emphasize the importance of continuous surveillance and antibiotic stewardship programs to guide effective treatment strategies for melioidosis (Table 1).

### 2.3. Quality of the Included Studies

The included studies showed variability in methodological quality, as shown in Supplementary File S1. Out of the 12 included studies, 2 studies were classified as high quality [10,16], whereas the 10 remaining studies were classified as moderate quality (11–16 points) [7,8,9,11,12,13,14,15,17,18], and no studies were categorized as low quality (<11 points).

### 2.4. Pool Prevalence of Antibiotic Resistance

The pooled prevalence of antibiotic resistance in *Burkholderia pseudomallei* varied across different antibiotics, as shown in Table 2, with the highest resistance observed for tigecycline (46.3%) and ciprofloxacin (38.3%), indicating potential limitations in their clinical efficacy. Moderate resistance was found for ceftriaxone (30.6%) and clavulanic acid (14.7%), while lower resistance rates were observed for ceftazidime (5.3%), trimethoprim–sulfamethoxazole (4.2%), doxycycline (1.7%), chloramphenicol (1.3%), and amoxicillin (0.48%). Imipenem and meropenem exhibited minimal resistance (0.09% and 0.08%, respectively).

Subgroup analysis revealed notable regional differences, with studies from Asia reporting higher resistance rates than those from America and Australia, as shown in Table 2. For instance, trimethoprim–sulfamethoxazole resistance was 5.5% in Asia and 4.1% in Australia, whereas no resistance was detected in America. Similarly, ciprofloxacin resistance (38.3%) was only reported in Asia. Resistance to ceftazidime also showed geographic variability, with 0.4% in Asia, 4.1% in Australia, and no detected resistance in America. When comparing testing methodologies, disk diffusion (DDF) consistently revealed higher resistance rates than minimum inhibitory concentration (MIC) testing. For example, resistance to trimethoprim–sulfamethoxazole was 47.0% using DDF but only 2.96% using MIC. Similarly, ceftazidime resistance was slightly higher using MIC (0.69%) compared to DDF (0.28%).

Statistical analysis indicated significant heterogeneity in resistance rates across studies by forest plot, as shown in Supplementary Figure S1. Ciprofloxacin (*I*^2^ = 91.3%), tigecycline (*I*^2^ = 91.2%), and chloramphenicol (*I*^2^ = 72.3%) exhibited high variability, suggesting inconsistencies in resistance detection between studies or differences in patient populations and local treatment practices. In contrast, resistance rates for clavulanic acid and ceftriaxone showed no significant variability (*I*^2^ = 0%), indicating more consistent resistance patterns. The statistical significance (*p* < 0.0001) for most antibiotics confirmed that these resistance trends were not due to random variation. The data are presented in Table 2 and Supplementary Figure S1.

## 3. Discussion

This study is the first systematic review and meta-analysis that evaluated the global resistance prevalence of *B. pseudomallei*, revealing varying levels among antibiotics used in the treatment of patients from Asia, Australia, and America. Twenty studies were included, highlighting the various antibiotics and resistance detection methodologies used across settings.

Melioidosis, a neglected tropical infectious disease, is caused by the Gram-negative bacterium *B. pseudomallei*. It exhibited intrinsic resistance to penicillin, ampicillin, first- and second-generation cephalosporins, gentamicin, tobramycin, and streptomycin. Currently, ceftazidime, imipenem, or meropenem are the preferred first-line intravenous drug treatments for acute or severe melioidosis according to the treatment guidelines from Thailand [19] and Australia [20]. Regarding the antibiotics used, the results from our systematic review and meta-analysis demonstrated that 22 antibiotics were used to eradicate *B. pseudomallei*. Eleven of them reported the incidence of drug-resistant isolates in varying proportions in various studies. Among these, tigecycline and ciprofloxacin demonstrated the highest pooled resistance rate, with 46.3 and 38.3%, respectively, indicating potential limitations in their clinical efficacy. Tigecycline is the first drug of the class in glycylcycline antibiotics and has a broad spectrum of activity against drug-resistant Gram-positive organisms. Tigecycline resistance generally occurs by alterations in tetracycline efflux or ribosomal protection [21]. However, resistances to tigecycline were reported only from two studies in Malaysia [7,12], suggesting that it is not a widely used antibiotic for melioidosis. Ciprofloxacin, a fluoroquinolone antibiotic, stops DNA replication by blocking the A subunit of DNA gyrase and having an extra impact on the substances in cell walls. Russell et al. (2000) demonstrated that despite using a susceptible strain of *B. pseudomallei* to determine efficacy by median lethal dose, ciprofloxacin was not effective when used therapeutically in vitro [22]. Ahmad et al. (2013) indicated that ciprofloxacin was less effective toward the *B. pseudomallei strain* [7]. Ceftazidime is a new ‘third-generation’ cephalosporin administered intravenously or intramuscularly. It exerts antibacterial activity by binding to penicillin-binding proteins and interfering with the synthesis of the bacterial cell wall, thus resulting in cell lysis and subsequent death. Since 1989, ceftazidime has been reported to reduce mortality in severe melioidosis by half [23] and has become the drug of choice for initial intensive therapy of melioidosis both in Asia and Australia [19,20]. However, pooled resistance rates were observed for 5.3% in our study, especially in Australia, and less in Asia. Antibiotics have been the most commonly used agents for eradication therapy, and it is to these that resistance has emerged in relapsed and persistent cases [11]. Combining trimethoprim with sulfamethoxazole agents, also known as co-trimoxazole, is meant to create a synergistic anti-folate effect; tetrahydrofolate is necessary for synthesizing purines required for DNA and protein production. On the other hand, trimethoprim–sulfamethoxazole (SXT) showed more resistance in Asia (5.5%) than in Australia (4.1%). However, the primary resistance of *B. pseudomallei* to SXT is extremely uncommon and should rarely be a contraindication to SXT monotherapy [24]. Paveenkittiporn et al. (2009) reported that 47% of cases from Thailand showed SXT resistance, but the susceptibility of *B. pseudomallei* to SXT determined by the disk diffusion method is unreliable; it must be performed using the minimal inhibitory concentration method [15]. SXT pooled resistance was predominated in Asia (1959/10,195 isolates; 19.2%) rather than Australia (7/170 isolates; 4.1%), as shown in our study. However, only one study from Australia and one from the USA were included in this analysis, whereas ten studies were from Asia. Thus, to avoid bias from geographical variation, more studies from other countries in the American and Australian continents should be included.

Imipenem and meropenem are broad-spectrum beta-lactam antibiotics, like other carbapenems, which bind to bacterial penicillin-binding proteins and interfere with bacterial cell wall integrity and synthesis. Our study showed that imipenem and meropenem exhibited minimal pooled resistance (0.09% and 0.08%, respectively). The two carbapenems (imipenem and meropenem) available in Australia consistently showed excellent antibiotic activity against *B*. *pseudomallei* [11]. A recent trial comparing imipenem and ceftazidime in severe melioidosis enrolled 214 culture-confirmed patients, with no difference in mortality between the two drugs. However, in the group of patients surviving at least 48 h, treatment failure was more common in those receiving ceftazidime [25]. It is therefore likely that imipenem or meropenem will be at least as effective as ceftazidime for initial intensive therapy of melioidosis [11].

The observed prevalence of antibiotic resistance also varied significantly by the method used for susceptibility testing. Two primary approaches were identified across the included studies: disk diffusion (DDF) and minimum inhibitory concentration (MIC)-based methods, including E-test, BMD (non-automation and automation). Our findings suggest that DDF consistently revealed higher resistance rates than MIC methods. For example, SXT resistance was reported at 47.0% using disk diffusion [15], but only 2.96% were identified using MIC techniques. A similar trend was noted for ceftazidime and tigecycline. This discrepancy is particularly concerning given the widespread use of disk diffusion in many resource-limited settings, where MIC methods are either unavailable or unaffordable. The reliability of disk diffusion for certain antibiotics—especially SXT—has been called into question by multiple studies. For *B. pseudomallei*, disk diffusion may underestimate zone sizes due to slow bacterial growth or overestimate resistance due to interpretive criteria not tailored to this organism. Paveenkittiporn et al. (2009) emphasized that SXT susceptibility testing should be confirmed with MIC, as disk diffusion results were often misleading [15]. Concordantly, Chea et al. (2024) demonstrated discrepancies in SXT susceptibility results when comparing disk diffusion and E-test with automated BMD [26]. Automated systems such as VITEK 2™ and Sensititre™, used in some studies [10,14], offer standardized, reproducible results but may also vary depending on local calibration and interpretive breakpoints. While BMD remains the gold standard for AST, its routine implementation in endemic regions remains limited due to cost, labor intensity, and technical expertise required. These methodological disparities highlight the urgent need for standardized testing protocols for *B. pseudomallei* globally. International guidelines should prioritize the harmonization of AST methods and encourage the validation of disk diffusion results with MIC, especially for critical drugs like SXT. Additionally, training and resource support should be expanded in endemic, low-resource settings to enable the adoption of reliable testing methods.

For many antibiotics, including trimethoprim–sulfamethoxazole (SXT) and ceftazidime (CAZ), MIC interpretive standards have been established in local or regional guidelines, such as those developed by research groups in Thailand and Australia [19,20]. These are often used in endemic areas due to the clinical need for tailored treatment approaches. For example, studies have reported SXT resistance using CLSI *P. aeruginosa* breakpoints (≤2/38 µg/mL for susceptible), although such interpretive thresholds may not align with actual clinical efficacy in melioidosis [24,26]. Furthermore, disk diffusion, which remains widely used in resource-limited endemic settings, lacks validated zone diameter breakpoints for *B. pseudomallei* in both CLSI and EUCAST guidelines. This has led to inconsistencies in resistance classification, especially for drugs like SXT and tigecycline, which are sensitive to methodological discrepancies [15,26]. Inaccurate interpretation due to inappropriate cut-off use can lead to either the underestimation or overestimation of resistance, affecting both treatment decisions and epidemiological data. To improve consistency in future studies and clinical practice, there is a need for international consensus and species-specific breakpoints for *B. pseudomallei*. Establishing validated MIC and disk diffusion thresholds, ideally informed by pharmacokinetic/pharmacodynamic (PK/PD) modeling and clinical outcomes, would allow for more accurate resistance surveillance and guide appropriate antimicrobial therapy.

There are some limitations to our report. These include the uneven geographic distribution of the included studies, with most data derived from Southeast Asia—particularly Thailand and Malaysia—while studies from other regions, such as Africa, South America, and parts of Oceania were lacking. This may limit the generalizability of our findings to a truly global context. Another limitation is the variation in antimicrobial susceptibility testing methods used across studies. Disk diffusion, although widely used in low-resource settings, is known to be less reliable for certain antibiotics like trimethoprim-sulfamethoxazole when compared to MIC-based methods, potentially leading to overestimation of resistance rates [15,26]. Additionally, publication bias may have occurred, as studies reporting resistance are more likely to be published than those showing no resistance. The quality and sample sizes of the included studies also varied, which may influence the robustness of the pooled estimates. Lastly, our analysis was limited to microbiological resistance data, as clinical outcome measures such as treatment failure or relapse were not consistently reported and therefore could not be analyzed. These limitations should be considered when interpreting our findings and highlight the need for more standardized, high-quality studies from diverse geographic regions.

## 4. Materials and Methods

### 4.1. Protocol and Registration

This systematic review was undertaken and reported following current methodological standards and the PRISMA (Preferred Reporting Items for Systematic Reviews and Meta-Analyses) guidelines [27]. The study protocol was registered with PROSPERO with registration number CRD420251015704.

### 4.2. Search Strategy and Study Selection

A systematic review was conducted using PubMed, Scopus, and Embase databases for studies published from database inception to December 2024. Keywords included “*Burkholderia pseudomallei*”, “antibiotic resistance”, and “drug resistance” combined with Boolean operators. Only studies published in English were included in the review. Studies were eligible if they reported on the prevalence of antibiotic-resistant *Burkholderia pseudomallei*, provided clear quantitative data on resistance rates, and were original research articles. Case reports, review articles, and those lacking full-text availability were excluded. Articles focusing on bacterial species other than *B. pseudomallei* were not considered. Furthermore, studies investigating in vitro, herbal, animal studies, or environmental resistance rather than clinical data were excluded. Finally, the meta-analysis was conducted for antibiotic resistance reports based on data from more than one study.

### 4.3. Data Extraction and Quality Assessment

Relevant data included author, year, country, sample size, methods of antibiotic susceptibility testing, and resistance prevalence for various antibiotics. Two independent reviewers extracted data from the included studies (J.T. and W.K.K.). Discrepancies were resolved through discussion or consultation with a third reviewer. The quality of the included studies was assessed using the Strengthening the Reporting of Observational Studies in Epidemiology (STROBE) checklist [28]. This tool evaluates the completeness and transparency of reporting in observational studies across 22 criteria, including study objectives, methodology, data collection, statistical analysis, and interpretation of findings. Each study received a score based on compliance with the checklist items. Scores were categorized as follows: high (≥17), moderate (11–16), or low quality (<11). Studies with higher scores demonstrated more rigorous methodological reporting, while moderate-quality studies showed minor reporting deficiencies but were still considered reliable for inclusion in the meta-analysis.

### 4.4. Data Analysis

Meta-analysis was performed using a random-effect model to estimate the pooled prevalence of antibiotic resistance. The *I*^2^ statistic was used for heterogeneity assessment, which was significant when the *p*-value was less than 0.05. Subgroup analyses were conducted based on geographic regions (Asia, Australia, and America) and testing methodology (MIC and disk diffusion). Forest plots were used to depict the study-specific proportions with 95% exact confidence intervals and overall pooled estimates with 95% Wald confidence intervals. All analyses were performed using R software version 4.4.3 with the ‘meta’ package (R Foundation for Statistical Computing, Vienna, Austria). For the meta-analyses of proportion studies with low proportion outcomes, funnel plots were not used, as they are ineffective at detecting potential publication bias [29]. Therefore, a publication bias assessment was not performed in the present study.

## 5. Conclusions

This systematic review and meta-analysis provide an updated synthesis of antibiotic resistance patterns in clinical isolates of *Burkholderia pseudomallei* globally. Our findings highlight significant variation in resistance prevalence across regions and testing methodologies. Although carbapenems, particularly imipenem and meropenem, continue to show excellent in vitro efficacy with minimal resistance, concerning rates were observed for ciprofloxacin and tigecycline, especially in Southeast Asia. First-line agents such as ceftazidime and trimethoprim–sulfamethoxazole remain largely effective but have demonstrated notable resistance in some regions.

Differences in susceptibility testing methods also impacted resistance estimates, with disk diffusion often yielding higher resistance rates compared to MIC-based techniques. These methodological inconsistencies emphasize the urgent need for standardized, species-specific interpretive criteria and widespread adoption of reliable testing practices.

To ensure effective clinical management and containment of resistant strains, continued global surveillance, harmonization of susceptibility testing protocols, and investment in laboratory capacity—particularly in endemic areas—are critical. Further research is needed to monitor resistance trends and evaluate their clinical impact on treatment outcomes in melioidosis.

## Figures and Tables

**Table 1:** Characters and details of the 12 included studies.

Author, Year	Ref.	Country	Method	Test Number (Total 10,391)	Antibiotic Susceptibility Test (Resistance Number)
Ahmad, 2013	[7]	Malaysia	MIC (E-test)	170	AMC (1), CAZ (1), CHL (1), **CIP (98)**, DOX (1), IPM (1), MEM (0), SAM (1), SXT (17), TGC (60), TZP (0)
Bugrysheva, 2021	[8]	USA	MIC (BMD)	26	AMC (1), AMX (0), **ATM (26)**, AZA (25), CAZ (0), CHL (0), CLA (0), DOX (0), IPM (0), SMX (3), SUL (0), SXT (0), TCY (0)
Fen, 2021	[9]	Thailand	MIC (BMD)	1317	AMC (2), **CAZ (3)**, IPM (0), MEM (0), SXT (1)
Hui, 2022	[10]	China	MIC (VITEK 2)	45	CAZ (3), **CIP (5)**, IPM (0), MEM (0), SXT (5)
Jenney, 2001	[11]	Australia	MIC (BMD)	170	AMC (0), **CAZ (7)**, CHL (0), CRO (0), **DOX (7), IPM (7), MEM (7),** PIP (0), **SXT (7)**
Khosravi, 2014	[12]	Malaysia	MIC (E-test)	69	AMC (25), CAZ (4), CHL (12), CLA (14), DOX (6), IPM (7), MEM (6), SXT (13), **TGC (41)**
Nhung, 2019	[13]	Vietnam	MIC (BMD)	312	AMC (0), CAZ (0), DOZ (2), IPM (0), **SXT (34)**
Panya, 2016	[14]	Thailand	MIC (Sensititre)	85	CAZ (0), CIP (46), **CRO (78)**, IPM (0)
Paveenkittiporn, 2009	[15]	Thailand	Disk diffusion	4019	AMC (201), CAZ (60), CTS (80), IPM (60), MEM (80), **SXT (1,889)**
Sia, 2021	[16]	Malaysia	Disk diffusion	129	**GEN (45)**
Sribenjalux, 2022	[17]	Thailand	MIC (BMD)	28	CAZ (0), IPM (0), MEM (0), SXT (0)
Wuthiekanun, 2011	[18]	Thailand	Disk diffusion	4021	**AMC (2), CAZ (2)**, IPM (0), MEM (0),

AMC, amoxicillin/clavulanic acid; AMX, amoxicillin; ATM, aztreonam; AZA, aztreonam/avibactam; CAZ, ceftazidime; CHL, chloramphenicol; CIP, ciprofloxacin; CLA, clavulanic acid; CRO, ceftriaxone; CTS, cefoperazone/sulbactam; DOX, doxycycline; GEN, gentamicin; IPM, imipenem; MEM, meropenem; PIP, piperacillin; SAM, ampicillin/sulbactam; SMX, trimethoprim; SUL, sulfamethoxazole; SXT, trimethoprim–sulfamethoxazole; TCY, tetracycline; TGC, tigecycline; TZP, piperacillin/tazobactam; MIC, minimum inhibitory concentration. The bold letter is the highest resistant number of each study.

**Table 2:** Pool prevalence according to subgroup meta-analysis of 11 antibiotic-resistant isolates.

Antibiotic	*n* of Study (*n* of Isolates)	Prevalence (95% CI)	*I* ^2^	*p*-Value
**AMC**	**8 (10,104)**	**0.0048 (0.0006–0.0371)**	**95.7**	**<0.0001**
**Region**				0.4572
Asia	6 (9908)	0.0054 (0.0005–0.0565)	96.9	<0.0001
America	1 (26)	0.0385 (0.0010–0.1964)		
Australia	1 (170)	0.0000 (0.0000–0.0215)		
**Method of testing**				0.9627
MIC	6 (2064)	0.0046 (0.0003–0.0595)	93.7	<0.0001
DDF	2 (8040)	0.0050 (0.0002–0.1295)	97.7	<0.0001
**CAZ**	**11 (10,262)**	**0.053 (0.0014–0.0192)**	**82.2**	**<0.0001**
**Region**				0.0208
Asia	9 (10,066)	0.0043 (0.0010–0.0177)	83.6	<0.0001
America	1 (26)	0.0000 (0.0000–0.1323)		
Australia	1 (170)	0.0412 (0.0167–0.0830)		
**Method of testing**				0.5343
MIC	9 (2222)	0.0069 (0.0016–0.0289)	70.6	0.0007
DDF	2 (8040)	0.0028 (0.0002–0.0329)	95.6	<0.0001
**CHL**	**4 (435)**	**0.0130 (0.0007–0.1992)**	**72.3**	**0.0107**
**Region**				1.0000
Asia	2 (239)	0.0797 (0.0247–0.2287)	91.1	0.0008
America	1 (26)	0.0000 (0.0000–0.1323)		
Australia	1 (170)	0.0000 (0.0000–0.0215)		
**Method of testing**				NA
MIC	4 (435)	0.0130 (0.0007–0.1992)	72.3	0.0107
**CIP**	**3 (300)**	**0.3826 (0.1562–0.6747)**	**91.3**	**<0.0001**
**Region**				NA
Asia	3 (300)	0.3826 (0.1562–0.6747)	91.3	<0.0001
**Method of testing**				NA
MIC	3 (300)	0.3826 (0.1562–0.6747)	91.3	<0.0001
**CLA**	**2 (95)**	**0.1474 (0.0893–0.2336) ^a^**	**0**	**0.9996**
**Region**			0	0.9996
Asia	1 (69)	0.0000 (0.0000–0.3169)		
America	1 (26)	0.0000 (0.0000–0.1323)		
**Method of testing**				NA
MIC	2 (95)	0.1474 (0.0893–0.2336) ^a^	0	0.9996
**CRO**	**2 (255)**	**0.3059 (0.2524–0.3652) ^a^**	**0**	**0.9995**
**Region**				0.9995
Asia	1 (85)	0.9176 (0.8377–0.9662)		
Australia	1 (170)	0.0000 (0.0000–0.0215)		
**Method of testing**				NA
MIC	2 (255)	0.3059 (0.2524–0.3652) ^a^	0	0.9995
**DOX**	**5 (747)**	**0.0174 (0.0052–0.0563)**	**72.2**	**0.0061**
**Region**				0.5283
Asia	3 (551)	0.0149 (0.0029–0.0726)	86	0.0008
America	1 (26)	0.0000 (0.0000–0.1323)		
Australia	1 (170)	0.0412 (0.0167–0.0830)		
**Method of testing**				NA
MIC	5 (747)	0.0174 (0.0052–0.0563)	72.2	0.0061
**IPM**	**11 (10,262)**	**0.0009 (0.0000–0.0161)**	**65.1**	**0.0014**
**Region**				<0.0001
Asia	9 (10,066)	0.0004 (0.0004–0.0004)	67	0.0021
America	1 (26)	0.0000 (0.0000–0.1323)		
Australia	1 (170)	0.0412 (0.0167–0.830)		
**Method of testing**				0.7273
MIC	9 (2222)	0.0012 (0.0000–0.0291) ^a^	6.7	0.3793
DDF	2 (8040)	0.0075 (0.0058–0.0096) ^a^	0	0.9996
**MEM**	**8 (9839)**	**0.0008 (0.0000–0.0341)**	**52.8**	**0.0382**
**Region**				0.0587
Asia	7 (9669)	0.0002 (0.0000–0.0473)	51	0.0568
Australia	1 (170)			
**Method of testing**				0.2825
MIC	6 (1799)	0.0072 (0.0042–0.0124) ^a^	0	0.861
DDF	2 (8040)	0.0100 (0.0080–0.0124) ^a^	0	0.9996
**SXT**	**9 (6156)**	**0.0423 (0.0097–0.1660)**	**97.4**	**<0.0001**
**Region**				0.953
Asia	7 (5960)	0.0547 (0.0120–0.2463)	97.7	<0.0001
America	1 (26)	0.0000 (0.0000–0.1323)		
Australia	1 (170)	0.0412 (0.067–0.0830)		
**Method of testing**				<0.0001
MIC	8 (2137)	0.0296 (0.0069–0.1175)	81.1	<0.0001
DDF	1 (4019)	0.47000 (0.4525–0.4856)		
**TGC**	**2 (239)**	**0.4634 (0.3025–0.6323)**	**91.2**	**0.0008**
**Region**				NA
Asia	2 (239)	0.4634 (0.3025–0.6323)	91.2	0.0008
**Method of testing**				NA
MIC	2 (239)	0.4634 (0.3025–0.6323)	91.2	0.0008

AMC, amoxicillin/clavulanic acid; CAZ, ceftazidime; CHL, chloramphenicol; CIP, ciprofloxacin; CLA, clavulanic acid; CRO, ceftriaxone; DOX, doxycycline; IPM, imipenem; MEM, meropenem; SXT, trimethoprim–sulfamethoxazole; TGC, tigecycline; MIC, minimum inhibitory concentration; DDF, disk diffusion; ^a^ calculated by a fixed mixed-effect model.

## Data Availability

No new data were created or analyzed in this study. Data sharing is not applicable to this article.

## Supplementary Materials

The following supporting information can be downloaded at: https://www.mdpi.com/article/10.3390/antibiotics14070647/s1, Supplementary File S1: STROBE Checklist; Supplementary Figure S1: Forest plots; and PRISMA 2020 checklist file.

## Abbreviations

The following abbreviations are used in this manuscript:

AMC	Amoxicillin/clavulanic acid
AMX	Amoxicillin
AST	Antimicrobial susceptibility testing
ATM	Aztreonam
AZA	Aztreonam/avibactam
BMD	Broth microdilution
CAZ	Ceftazidime
CHL	Chloramphenicol
CIP	Ciprofloxacin
CLA	Clavulanic acid
CRO	Ceftriaxone
CTS	Cefoperazone/sulbactam
DOX	Doxycycline
DDF	Disk diffusion
IPM	Imipenem
MEM	Meropenem
MIC	Minimum inhibitory concentration
PIP	Piperacillin
SAM	Ampicillin/sulbactam
SMX	Trimethoprim
SUL	Sulfamethoxazole
SXT	Trimethoprim–sulfamethoxazole
TCY	Tetracycline
TGC	Tigecycline
TZP	Piperacillin/tazobactam
