# Supplementary material for: Global Prevalence of Antibiotic-Resistant Burkholderia pseudomallei in Melioidosis Patients: A Systematic Review and Meta-Analysis
Source: Antibiotics (Basel). 2025 Jun 25;14(7):647. doi: 10.3390/antibiotics14070647 (PMC12291811; doi:10.3390/antibiotics14070647)
Supplement: Supplementary file 1 [file antibiotics-14-00647-s001.zip › Supplementary Figure 1.pdf]

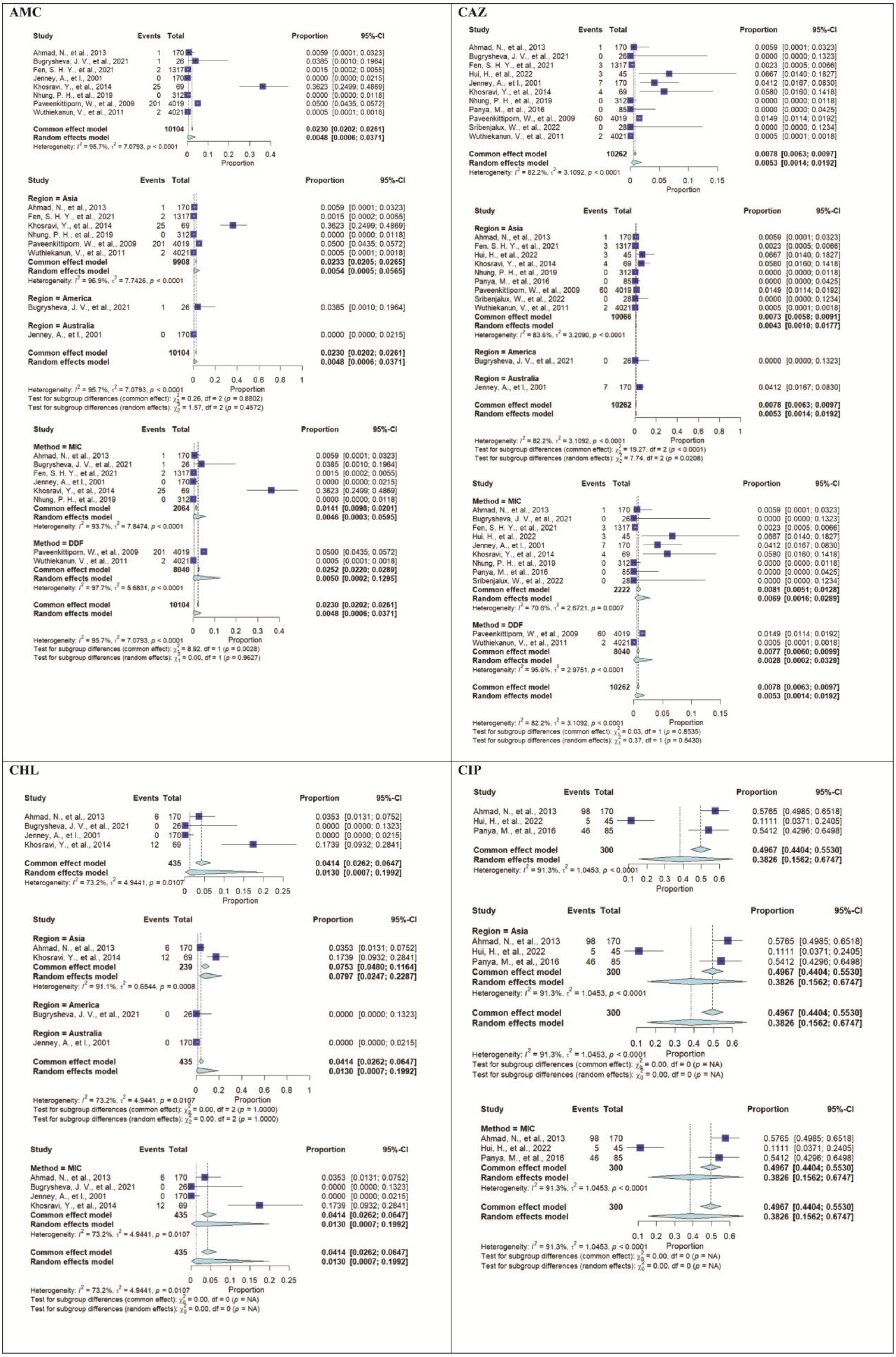

AMC
Study Events Total Proportion 95%-CI
Ahmad, N., et al., 2013 1 170 0.0059 [0.0001; 0.0323]
Bugrysheva, J. V., et al., 2021 1 26 0.0385 [0.0010; 0.1964]
Fen, S. H. Y., et al., 2021 2 1317 0.0015 [0.0002; 0.0055]
Jenney, A., et l., 2001 0 170 0.0000 [0.0000; 0.0215]
Khosravi, Y., et al., 2014 25 69 0.3623 [0.2499; 0.4869]
Nhung, P. H., et al., 2019 0 312 0.0000 [0.0000; 0.0118]
Paveenkittiporn, W., et al., 2009 201 4019 0.0500 [0.0435; 0.0572]
Wuthiekanun, V., et al., 2011 2 4021 0.0005 [0.0001; 0.0018]
Common effect model 10104 0.0230 [0.0202; 0.0261]
Random effects model 0.0048 [0.0006; 0.0371]
Heterogeneity: I^2 = 95.7%, τ^2 = 7.0793, p < 0.0001
0 0.1 0.2 0.3 0.4
Proportion
Study Events Total Proportion 95%-CI
Region = Asia
Ahmad, N., et al., 2013 1 170 0.0059 [0.0001; 0.0323]
Fen, S. H. Y., et al., 2021 2 1317 0.0015 [0.0002; 0.0055]
Khosravi, Y., et al., 2014 25 69 0.3623 [0.2499; 0.4869]
Nhung, P. H., et al., 2019 0 312 0.0000 [0.0000; 0.0118]
Paveenkittiporn, W., et al., 2009 201 4019 0.0500 [0.0435; 0.0572]
Wuthiekanun, V., et al., 2011 2 4021 0.0005 [0.0001; 0.0018]
Common effect model 9908 0.0233 [0.0205; 0.0265]
Random effects model 0.0054 [0.0005; 0.0565]
Heterogeneity: I^2 = 96.9%, τ^2 = 7.7426, p < 0.0001
Region = America
Bugrysheva, J. V., et al., 2021 1 26 0.0385 [0.0010; 0.1964]
Region = Australia
Jenney, A., et l., 2001 0 170 0.0000 [0.0000; 0.0215]
Common effect model 10104 0.0230 [0.0202; 0.0261]
Random effects model 0.0048 [0.0006; 0.0371]
0 0.2 0.4 0.6 0.8 1
Proportion
Heterogeneity: I^2 = 95.7%, τ^2 = 7.0793, p < 0.0001
Test for subgroup differences (common effect): χ^2_2 = 0.26, df = 2 (p = 0.8802)
Test for subgroup differences (random effects): χ^2_2 = 1.57, df = 2 (p = 0.4572)
Study Events Total Proportion 95%-CI
Method = MIC
Ahmad, N., et al., 2013 1 170 0.0059 [0.0001; 0.0323]
Bugrysheva, J. V., et al., 2021 1 26 0.0385 [0.0010; 0.1964]
Fen, S. H. Y., et al., 2021 2 1317 0.0015 [0.0002; 0.0055]
Jenney, A., et l., 2001 0 170 0.0000 [0.0000; 0.0215]
Khosravi, Y., et al., 2014 25 69 0.3623 [0.2499; 0.4869]
Nhung, P. H., et al., 2019 0 312 0.0000 [0.0000; 0.0118]
Common effect model 2064 0.0141 [0.0098; 0.0201]
Random effects model 0.0046 [0.0003; 0.0595]
Heterogeneity: I^2 = 93.7%, τ^2 = 7.8474, p < 0.0001
Method = DDF
Paveenkittiporn, W., et al., 2009 201 4019 0.0500 [0.0435; 0.0572]
Wuthiekanun, V., et al., 2011 2 4021 0.0005 [0.0001; 0.0018]
Common effect model 8040 0.0252 [0.0220; 0.0289]
Random effects model 0.0050 [0.0002; 0.1295]
Heterogeneity: I^2 = 97.7%, τ^2 = 5.6831, p < 0.0001
Common effect model 10104 0.0230 [0.0202; 0.0261]
Random effects model 0.0048 [0.0006; 0.0371]
0 0.1 0.2 0.3 0.4
Proportion
Heterogeneity: I^2 = 95.7%, τ^2 = 7.0793, p < 0.0001
Test for subgroup differences (common effect): χ^2_1 = 8.92, df = 1 (p = 0.0028)
Test for subgroup differences (random effects): χ^2_1 = 0.00, df = 1 (p = 0.9627)
CAZ
Study Events Total Proportion 95%-CI
Ahmad, N., et al., 2013 1 170 0.0059 [0.0001; 0.0323]
Bugrysheva, J. V., et al., 2021 0 26 0.0000 [0.0000; 0.1323]
Fen, S. H. Y., et al., 2021 3 1317 0.0023 [0.0005; 0.0066]
Hui, H., et al., 2022 3 45 0.0667 [0.0140; 0.1827]
Jenney, A., et l., 2001 7 170 0.0412 [0.0167; 0.0830]
Khosravi, Y., et al., 2014 4 69 0.0580 [0.0160; 0.1418]
Nhung, P. H., et al., 2019 0 312 0.0000 [0.0000; 0.0118]
Panya, M., et al., 2016 0 85 0.0000 [0.0000; 0.0425]
Paveenkittiporn, W., et al., 2009 60 4019 0.0149 [0.0114; 0.0192]
Sribenjalux, W., et al., 2022 0 28 0.0000 [0.0000; 0.1234]
Wuthiekanun, V., et al., 2011 2 4021 0.0005 [0.0001; 0.0018]
Common effect model 10262 0.0078 [0.0063; 0.0097]
Random effects model 0.0053 [0.0014; 0.0192]
Heterogeneity: I^2 = 82.2%, τ^2 = 3.1092, p < 0.0001
0 0.05 0.1 0.15
Proportion
Study Events Total Proportion 95%-CI
Region = Asia
Ahmad, N., et al., 2013 1 170 0.0059 [0.0001; 0.0323]
Fen, S. H. Y., et al., 2021 3 1317 0.0023 [0.0005; 0.0066]
Hui, H., et al., 2022 3 45 0.0667 [0.0140; 0.1827]
Khosravi, Y., et al., 2014 4 69 0.0580 [0.0160; 0.1418]
Nhung, P. H., et al., 2019 0 312 0.0000 [0.0000; 0.0118]
Panya, M., et al., 2016 0 85 0.0000 [0.0000; 0.0425]
Paveenkittiporn, W., et al., 2009 60 4019 0.0149 [0.0114; 0.0192]
Sribenjalux, W., et al., 2022 0 28 0.0000 [0.0000; 0.1234]
Wuthiekanun, V., et al., 2011 2 4021 0.0005 [0.0001; 0.0018]
Common effect model 10066 0.0073 [0.0058; 0.0091]
Random effects model 0.0043 [0.0010; 0.0177]
Heterogeneity: I^2 = 83.6%, τ^2 = 3.2090, p < 0.0001
Region = America
Bugrysheva, J. V., et al., 2021 0 26 0.0000 [0.0000; 0.1323]
Region = Australia
Jenney, A., et l., 2001 7 170 0.0412 [0.0167; 0.0830]
Common effect model 10262 0.0078 [0.0063; 0.0097]
Random effects model 0.0053 [0.0014; 0.0192]
0 0.2 0.4 0.6 0.8 1
Proportion
Heterogeneity: I^2 = 82.2%, τ^2 = 3.1092, p < 0.0001
Test for subgroup differences (common effect): χ^2_2 = 19.27, df = 2 (p < 0.0001)
Test for subgroup differences (random effects): χ^2_2 = 7.74, df = 2 (p = 0.0208)
Study Events Total Proportion 95%-CI
Method = MIC
Ahmad, N., et al., 2013 1 170 0.0059 [0.0001; 0.0323]
Bugrysheva, J. V., et al., 2021 0 26 0.0000 [0.0000; 0.1323]
Fen, S. H. Y., et al., 2021 3 1317 0.0023 [0.0005; 0.0066]
Hui, H., et al., 2022 3 45 0.0667 [0.0140; 0.1827]
Jenney, A., et l., 2001 7 170 0.0412 [0.0167; 0.0830]
Khosravi, Y., et al., 2014 4 69 0.0580 [0.0160; 0.1418]
Nhung, P. H., et al., 2019 0 312 0.0000 [0.0000; 0.0118]
Panya, M., et al., 2016 0 85 0.0000 [0.0000; 0.0425]
Sribenjalux, W., et al., 2022 0 28 0.0000 [0.0000; 0.1234]
Common effect model 2222 0.0081 [0.0051; 0.0128]
Random effects model 0.0069 [0.0016; 0.0289]
Heterogeneity: I^2 = 70.6%, τ^2 = 2.6721, p = 0.0007
Method = DDF
Paveenkittiporn, W., et al., 2009 60 4019 0.0149 [0.0114; 0.0192]
Wuthiekanun, V., et al., 2011 2 4021 0.0005 [0.0001; 0.0018]
Common effect model 8040 0.0077 [0.0060; 0.0099]
Random effects model 0.0028 [0.0002; 0.0329]
Heterogeneity: I^2 = 95.6%, τ^2 = 2.9751, p < 0.0001
Common effect model 10262 0.0078 [0.0063; 0.0097]
Random effects model 0.0053 [0.0014; 0.0192]
0 0.05 0.1 0.15
Proportion
Heterogeneity: I^2 = 82.2%, τ^2 = 3.1092, p < 0.0001
Test for subgroup differences (common effect): χ^2_1 = 0.03, df = 1 (p = 0.8535)
Test for subgroup differences (random effects): χ^2_1 = 0.37, df = 1 (p = 0.5430)
CHL
Study Events Total Proportion 95%-CI
Ahmad, N., et al., 2013 6 170 0.0353 [0.0131; 0.0752]
Bugrysheva, J. V., et al., 2021 0 26 0.0000 [0.0000; 0.1323]
Jenney, A., et l., 2001 0 170 0.0000 [0.0000; 0.0215]
Khosravi, Y., et al., 2014 12 69 0.1739 [0.0932; 0.2841]
Common effect model 435 0.0414 [0.0262; 0.0647]
Random effects model 0.0130 [0.0007; 0.1992]
Heterogeneity: I^2 = 73.2%, τ^2 = 4.9441, p = 0.0107
0 0.05 0.1 0.15 0.2 0.25
Proportion
Study Events Total Proportion 95%-CI
Region = Asia
Ahmad, N., et al., 2013 6 170 0.0353 [0.0131; 0.0752]
Khosravi, Y., et al., 2014 12 69 0.1739 [0.0932; 0.2841]
Common effect model 239 0.0753 [0.0480; 0.1164]
Random effects model 0.0797 [0.0247; 0.2287]
Heterogeneity: I^2 = 91.1%, τ^2 = 0.6544, p = 0.0008
Region = America
Bugrysheva, J. V., et al., 2021 0 26 0.0000 [0.0000; 0.1323]
Region = Australia
Jenney, A., et l., 2001 0 170 0.0000 [0.0000; 0.0215]
Common effect model 435 0.0414 [0.0262; 0.0647]
Random effects model 0.0130 [0.0007; 0.1992]
0 0.2 0.4 0.6 0.8 1
Proportion
Heterogeneity: I^2 = 73.2%, τ^2 = 4.9441, p = 0.0107
Test for subgroup differences (common effect): χ^2_2 = 0.00, df = 2 (p = 1.0000)
Test for subgroup differences (random effects): χ^2_2 = 0.00, df = 2 (p = 1.0000)
Study Events Total Proportion 95%-CI
Method = MIC
Ahmad, N., et al., 2013 6 170 0.0353 [0.0131; 0.0752]
Bugrysheva, J. V., et al., 2021 0 26 0.0000 [0.0000; 0.1323]
Jenney, A., et l., 2001 0 170 0.0000 [0.0000; 0.0215]
Khosravi, Y., et al., 2014 12 69 0.1739 [0.0932; 0.2841]
Common effect model 435 0.0414 [0.0262; 0.0647]
Random effects model 0.0130 [0.0007; 0.1992]
Heterogeneity: I^2 = 73.2%, τ^2 = 4.9441, p = 0.0107
Common effect model 435 0.0414 [0.0262; 0.0647]
Random effects model 0.0130 [0.0007; 0.1992]
0 0.05 0.1 0.15 0.2 0.25
Proportion
Heterogeneity: I^2 = 73.2%, τ^2 = 4.9441, p = 0.0107
Test for subgroup differences (common effect): χ^2_0 = 0.00, df = 0 (p = NA)
Test for subgroup differences (random effects): χ^2_0 = 0.00, df = 0 (p = NA)
CIP
Study Events Total Proportion 95%-CI
Ahmad, N., et al., 2013 98 170 0.5765 [0.4985; 0.6518]
Hui, H., et al., 2022 5 45 0.1111 [0.0371; 0.2405]
Panya, M., et al., 2016 46 85 0.5412 [0.4296; 0.6498]
Common effect model 300 0.4967 [0.4404; 0.5530]
Random effects model 0.3826 [0.1562; 0.6747]
Heterogeneity: I^2 = 91.3%, τ^2 = 1.0453, p < 0.0001
0.1 0.2 0.3 0.4 0.5 0.6
Proportion
Study Events Total Proportion 95%-CI
Region = Asia
Ahmad, N., et al., 2013 98 170 0.5765 [0.4985; 0.6518]
Hui, H., et al., 2022 5 45 0.1111 [0.0371; 0.2405]
Panya, M., et al., 2016 46 85 0.5412 [0.4296; 0.6498]
Common effect model 300 0.4967 [0.4404; 0.5530]
Random effects model 0.3826 [0.1562; 0.6747]
Heterogeneity: I^2 = 91.3%, τ^2 = 1.0453, p < 0.0001
Common effect model 300 0.4967 [0.4404; 0.5530]
Random effects model 0.3826 [0.1562; 0.6747]
0.1 0.2 0.3 0.4 0.5 0.6
Proportion
Heterogeneity: I^2 = 91.3%, τ^2 = 1.0453, p < 0.0001
Test for subgroup differences (common effect): χ^2_0 = 0.00, df = 0 (p = NA)
Test for subgroup differences (random effects): χ^2_0 = 0.00, df = 0 (p = NA)
Study Events Total Proportion 95%-CI
Method = MIC
Ahmad, N., et al., 2013 98 170 0.5765 [0.4985; 0.6518]
Hui, H., et al., 2022 5 45 0.1111 [0.0371; 0.2405]
Panya, M., et al., 2016 46 85 0.5412 [0.4296; 0.6498]
Common effect model 300 0.4967 [0.4404; 0.5530]
Random effects model 0.3826 [0.1562; 0.6747]
Heterogeneity: I^2 = 91.3%, τ^2 = 1.0453, p < 0.0001
Common effect model 300 0.4967 [0.4404; 0.5530]
Random effects model 0.3826 [0.1562; 0.6747]
0.1 0.2 0.3 0.4 0.5 0.6
Proportion
Heterogeneity: I^2 = 91.3%, τ^2 = 1.0453, p < 0.0001
Test for subgroup differences (common effect): χ^2_0 = 0.00, df = 0 (p = NA)
Test for subgroup differences (random effects): χ^2_0 = 0.00, df = 0 (p = NA)

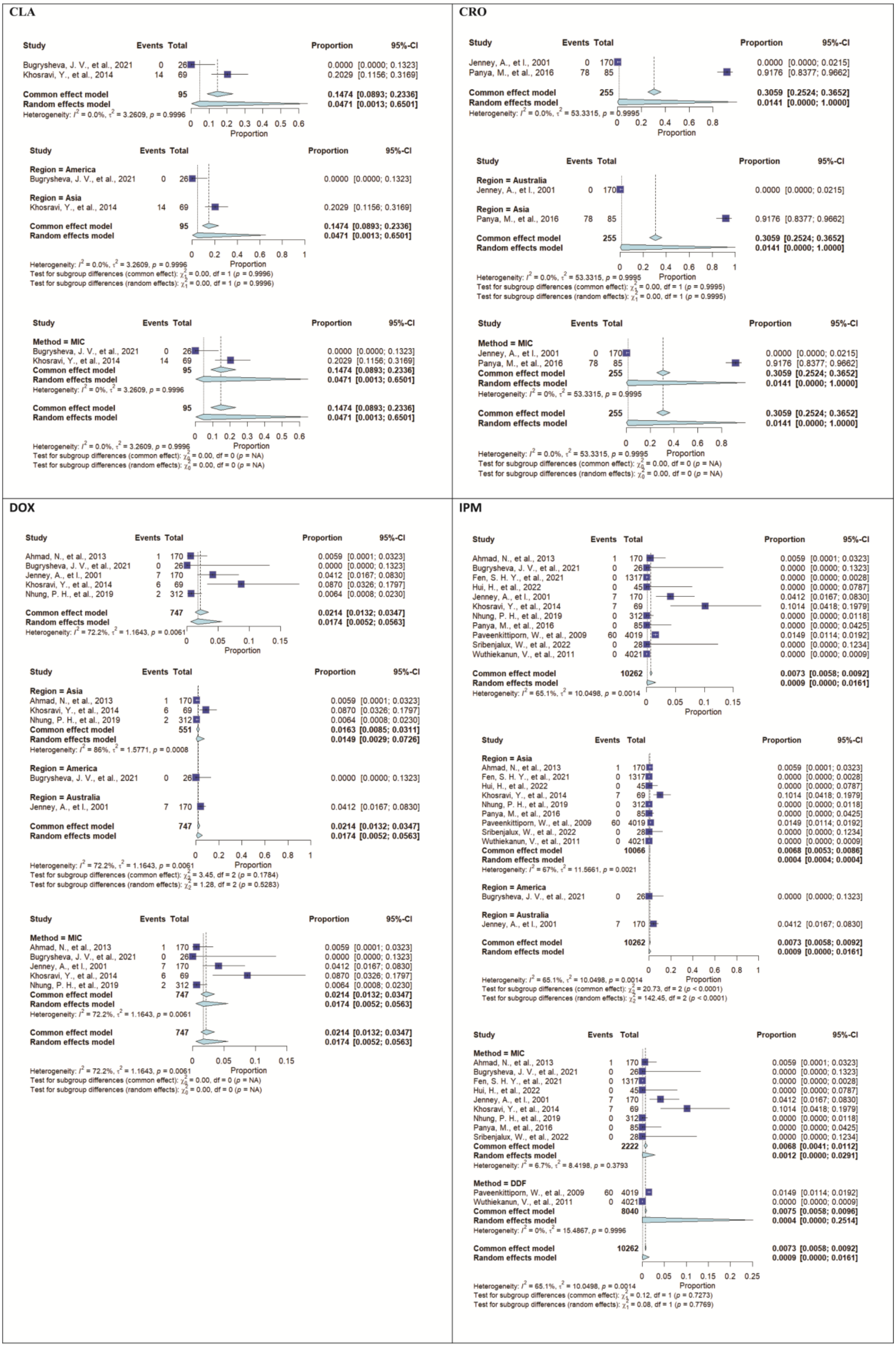

CLA
Study Events Total Proportion 95%-CI
Bugrysheva, J. V., et al., 2021 0 26 0.0000 [0.0000; 0.1323]
Khosravi, Y., et al., 2014 14 69 0.2029 [0.1156; 0.3169]
Common effect model 95 0.1474 [0.0893; 0.2336]
Random effects model 0.0471 [0.0013; 0.6501]
Proportion
Region = America
Region = Asia
Method = MIC
CRO
Jenney, A., et l., 2001 0 170 0.0000 [0.0000; 0.0215]
Panya, M., et al., 2016 78 85 0.9176 [0.8377; 0.9662]
Common effect model 255 0.3059 [0.2524; 0.3652]
Random effects model 0.0141 [0.0000; 1.0000]
Region = Australia
DOX
Ahmad, N., et al., 2013 1 170 0.0059 [0.0001; 0.0323]
Bugrysheva, J. V., et al., 2021 0 26 0.0000 [0.0000; 0.1323]
Jenney, A., et l., 2001 7 170 0.0412 [0.0167; 0.0830]
Khosravi, Y., et al., 2014 6 69 0.0870 [0.0326; 0.1797]
Nhung, P. H., et al., 2019 2 312 0.0064 [0.0008; 0.0230]
Common effect model 747 0.0214 [0.0132; 0.0347]
Random effects model 0.0174 [0.0052; 0.0563]
IPM
Ahmad, N., et al., 2013 1 170 0.0059 [0.0001; 0.0323]
Bugrysheva, J. V., et al., 2021 0 26 0.0000 [0.0000; 0.1323]
Fen, S. H. Y., et al., 2021 0 1317 0.0000 [0.0000; 0.0028]
Hui, H., et al., 2022 0 45 0.0000 [0.0000; 0.0787]
Jenney, A., et l., 2001 7 170 0.0412 [0.0167; 0.0830]
Khosravi, Y., et al., 2014 7 69 0.1014 [0.0418; 0.1979]
Nhung, P. H., et al., 2019 0 312 0.0000 [0.0000; 0.0118]
Panya, M., et al., 2016 0 85 0.0000 [0.0000; 0.0425]
Paveenkittiporn, W., et al., 2009 60 4019 0.0149 [0.0114; 0.0192]
Sribenjalux, W., et al., 2022 0 28 0.0000 [0.0000; 0.1234]
Wuthiekanun, V., et al., 2011 0 4021 0.0000 [0.0000; 0.0009]
Common effect model 10262 0.0073 [0.0058; 0.0092]
Random effects model 0.0009 [0.0000; 0.0161]
Method = DDF

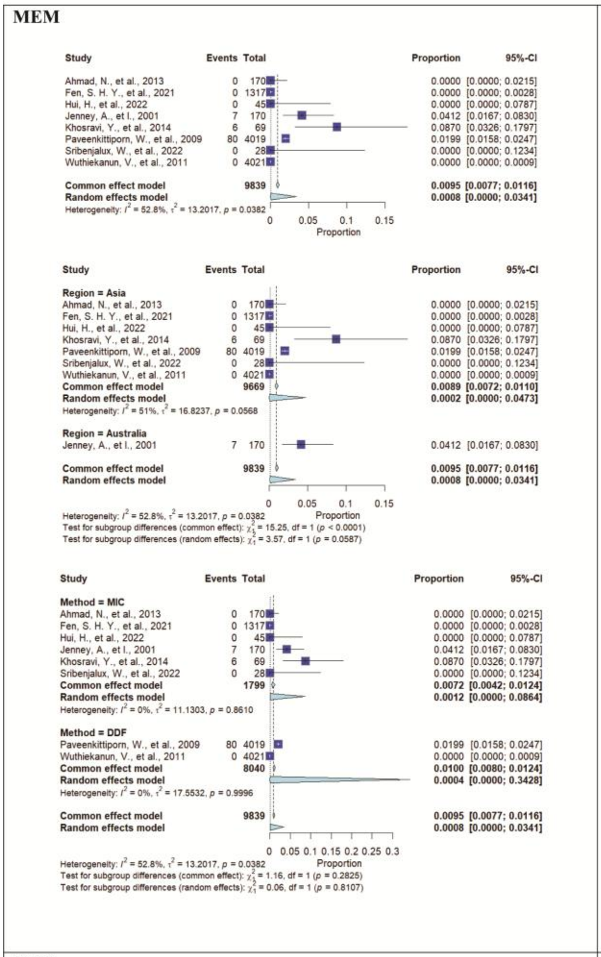

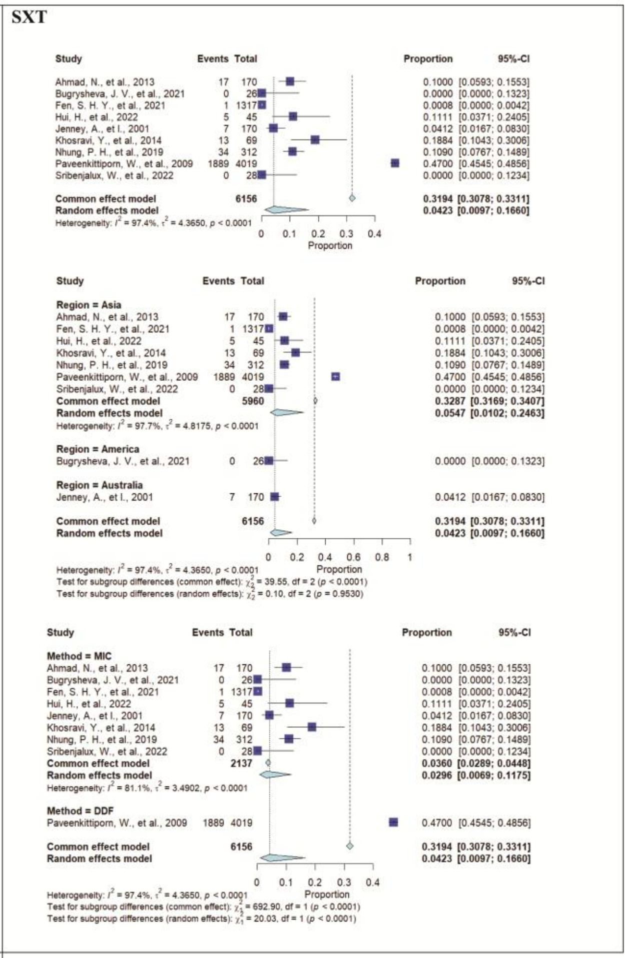

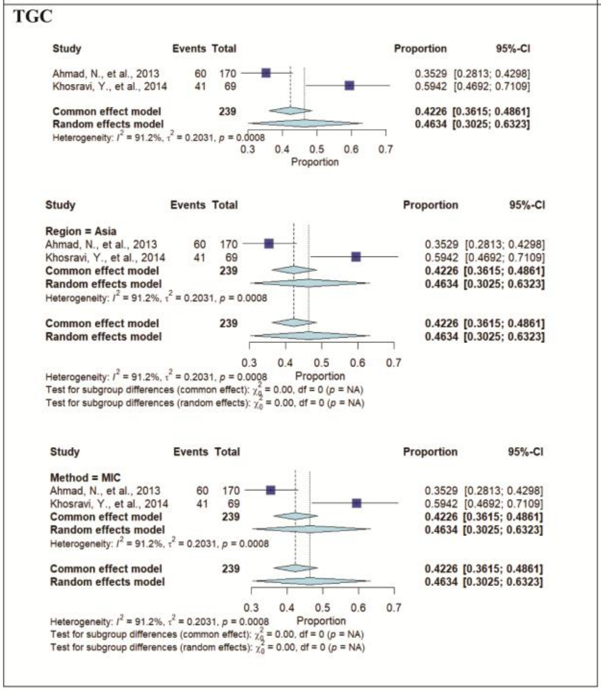

Supplementary Figure 1. Forest plots of 11 antibiotics-resistance and sub-groups analysis across difference regions and methods. AMC; amoxycillin/clavulanic acid, CAZ; ceftazidime, CHL; chloramphenicol, CIP; ciprofloxacin, CLA; clavulanic acid, CRO; Ceftriaxone, DOX; doxycycline, IPM; imipenem, MEM; meropenem, SXT; trimethoprim/sulfamethoxazole, TGC; tigecycline, MIC; minimum inhibitory concentration, DDF; disk diffusion.
